# Painless Aortic Dissection With Bradycardia, Hypotension, and Lower Limb Weakness: A Case Report

**DOI:** 10.7759/cureus.94540

**Published:** 2025-10-14

**Authors:** Kaho Sakurai, Ryoichi Inoue, Toshinori Nishizawa

**Affiliations:** 1 Emergency Department, Yokosuka General Medical Center, Yokosuka, JPN; 2 Internal Medicine, The Ohio State University Wexner Medical Center, Columbus, USA; 3 General Internal Medicine, St. Luke's International Hospital, Tokyo, JPN

**Keywords:** aortic dissection, bradycardia, carotid ultrasound, hypotension, neurological symptoms, painless presentation

## Abstract

Aortic dissection typically presents with chest or back pain, but it can be painless. In the absence of typical pain, diagnosis may be delayed or missed. We present a case of an 84-year-old woman in which the initial manifestations were transient bilateral lower-limb weakness accompanied by bradycardia and hypotension. Electrocardiography demonstrated marked sinus bradycardia without ischemic changes, and laboratory tests were unremarkable except for an elevated D-dimer. Bedside carotid duplex ultrasonography incidentally identified an intimal flap in the common carotid artery, and contrast-enhanced computed tomography (CECT) confirmed Stanford type A aortic dissection involving the aortic arch with extension into both common carotid arteries. The patient underwent emergent valve-sparing supracoronary ascending aortic replacement. This case underscores the importance of recognizing unexplained bradycardia, hypotension, and transient muscle weakness as early clues to painless aortic dissection. Carotid duplex ultrasonography may aid diagnosis in such atypical presentations and should be considered when standard evaluations are inconclusive, potentially reducing missed or delayed diagnoses.

## Introduction

Aortic dissection is an acute aortic syndrome in which an intimal tear creates true and false lumens and can rapidly progress to life-threatening complications. Its incidence is relatively low but increases with age, and hypertension is the most prominent risk factor. Although patients typically present with abrupt, severe chest or back pain, approximately 5-10% of cases are painless; in this subgroup, syncope and new-onset neurologic deficits are common initial manifestations, and missed or delayed diagnosis is not uncommon [1].

By the Stanford classification, type A involves the ascending aorta and generally requires emergent surgical management, whereas type B spares the ascending aorta and, if uncomplicated, is managed medically [2]. Despite advances in imaging and perioperative care, early mortality remains high, particularly for type A, so rapid diagnosis and definitive treatment are critical; in most settings, the diagnosis is confirmed by contrast-enhanced computed tomography (CECT) as the first-line imaging modality [3]. We report a painless Stanford type A dissection that initially manifested as transient bilateral lower-limb weakness accompanied by bradycardia and hypotension. Bedside carotid duplex ultrasonography identified an intimal flap, which prompted definitive CECT and expedited surgical management.

## Case presentation

An 84-year-old woman presented to the emergency department with sudden, transient bilateral lower-limb weakness lasting 90 minutes. She denied chest, neck, back, or abdominal pain. Her medical history included hypertension and hyperlipidemia, with no history of smoking or a family history of connective tissue disorders. Her medications included nilvadipine 2 mg (0.04 mg/kg), amlodipine 2.5 mg (0.05 mg/kg), atorvastatin 5 mg (0.10 mg/kg), and bezafibrate 200 mg (4.0 mg/kg).

**Figure 1:**
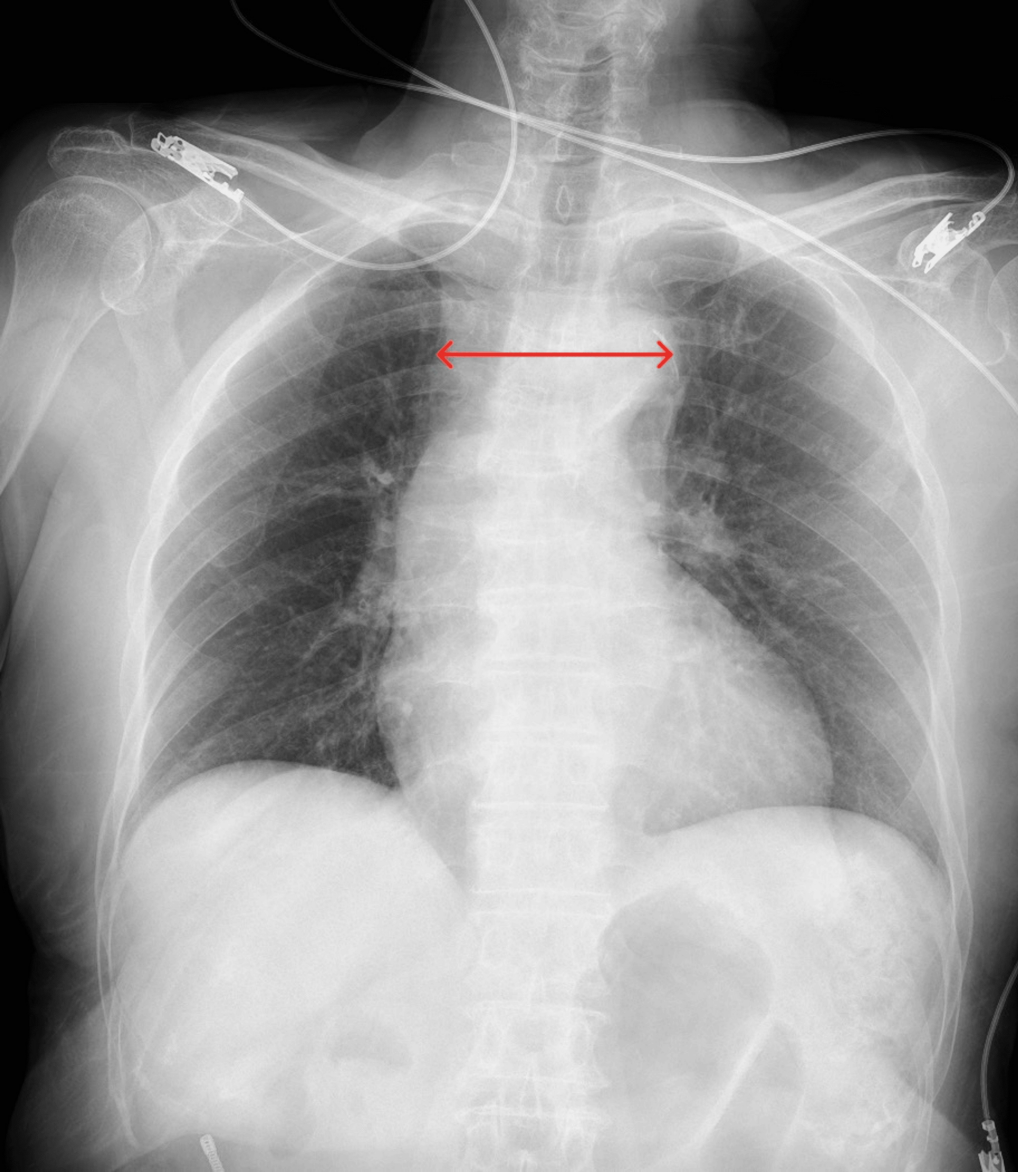
Chest radiograph at admission The red arrow shows an 83-mm upper mediastinal width, indicating mediastinal widening.

On examination, she was fully alert and oriented. Vital signs were as follows: blood pressure 84/38 mmHg in both arms, heart rate 44 beats/minute, respiratory rate 20 breaths/minute, body temperature 35.2°C (95.4°F), and oxygen saturation 97% on room air. Manual muscle testing of both lower extremities was 4/5, and the neurologic examination revealed no focal deficits. A chest radiograph showed a mildly widened mediastinum (Figure 1). Laboratory tests, including electrolytes, renal and liver function, and cardiac markers, were within normal limits. The white blood cell count was 5,800/µL, hemoglobin 11.6 g/dL, platelet count 234,000/µL, and D-dimer 5.4 µg/mL (Table 1).

**Table 1:** Laboratory test results PT, prothrombin pime; CK-MB, creatine kinase–MB isoenzyme; INR, International Normalized Ratio; aPTT, activated Partial Thromboplastin Time; BNP, B-type natriuretic peptide

Parameters	Patient value	Reference value
White Blood Cell Count	5800	3300-8600 cells/mm³
Hemoglobin	11.6	11.6-14.8 g/dL
Platelet Count	234000	158000-348000 cells/cm³
Total Protein	6.6	6.6-8.1 g/dL
Albumin	3.9	4.1-5.1 g/dL
Blood Urea Nitrogen	16.0	8-20 mg/dL
Serum Creatinine	1.06	0.46-0.79 mg/dL
Serum Sodium	143	138-145 mEq/L
Potassium	4.4	3.6-4.8 mEq/L
Bicarbonate	N/A	22-26 mmol/L
Total Bilirubin	0.43	0.4-1.5 mg/dL
Direct Bilirubin	0.13	< 0.4 mg/dL
Aspartate Aminotransferase	15	13-30 U/L
Alanine Aminotransferase	8	7-23 U/L
Gamma-Glutamyl Transpeptidase	14	< 32 U/L
Alkaline Phosphatase	44	38-113 U/L
Calcium	9.0	8.4–10.2 mg/dL
CK-MB	29	0–12 U/L
C-Reactive Protein	0.05	< 0.3 mg/dL
Glucose	115	73-109 mg/dL
Troponin	<10.0	Female <16 ng/L, Male <34 ng/L（99th percentile）
PT（sec）	10.4	10–13 s
PT (%)	103.6	70–130 %
PT-INR	0.98	0.9-1.1
APTT	20.5	24-40s
BNP	36.1	≤18.4 pg/mL
D-dimer	5.4	< 0.5 μg/mL

Electrocardiography demonstrated marked sinus bradycardia without ischemic changes, ST elevation, bundle-branch block, or PR/QT prolongation. Transthoracic echocardiography (TTE) showed normal chamber dimensions, a preserved left ventricular ejection fraction, no pathological aortic insufficiency, and no pericardial effusion. However, bedside carotid duplex ultrasonography revealed an intimal flap in the common carotid artery.

**Figure 2:**
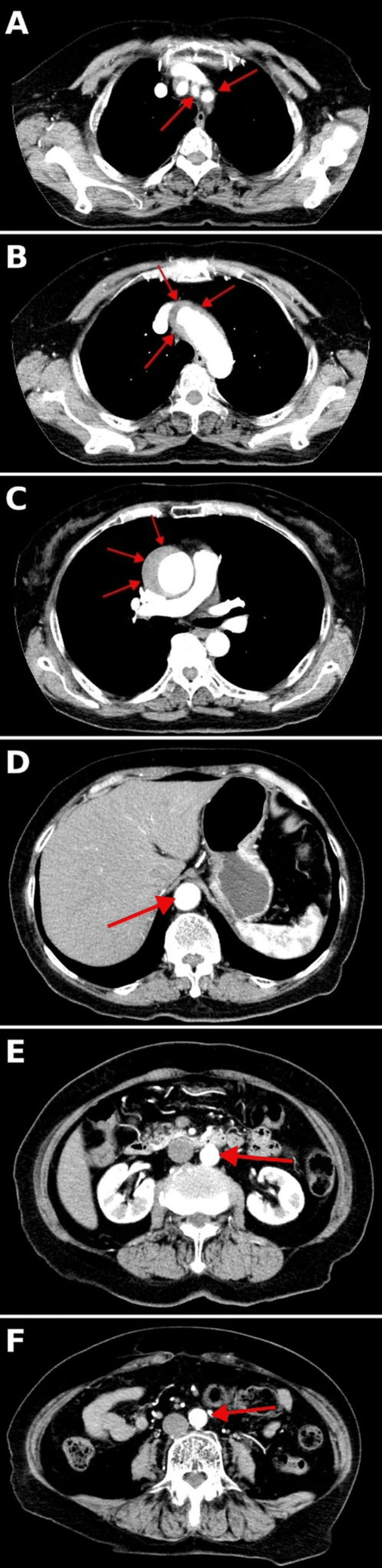
Axial contrast-enhanced computed tomography (A) Common carotid artery: intimal flap with a false lumen within the common carotid artery (red arrows); (B) Aortic arch: intimal flap with a false lumen in the arch (red arrows); (C) Aortic root/proximal ascending aorta: intimal flap with a false lumen abutting the aortic root (red arrow); (D–F) Descending thoracic to abdominal aorta: arrows indicate the aortic lumen; no intimal flap is visible at the supraceliac, infrarenal, and distal abdominal levels.

CECT confirmed a Stanford type A aortic dissection involving the aortic arch, with common carotid artery dissection and extension to the aortic root (Figure 2). She was admitted to the cardiovascular surgery intensive care unit and underwent supracoronary ascending aortic replacement with aortic valve resuspension using a Dacron tube graft (J-graft 26 mm with a 9 mm side branch). No aortic root replacement and no coronary button reimplantation were performed. Cerebral/neural protection consisted of systemic hypothermia and selective antegrade cerebral perfusion (SACP) via the brachiocephalic trunk and the left common carotid artery during circulatory arrest. The aortic cross-clamp time was 66 minutes, and the cardiopulmonary bypass time was 75 minutes. Temporary pacing was required preoperatively only. Her hypotension and bradycardia resolved postoperatively, and no residual limb weakness was observed. She was discharged without complications on hospital day 10.

At the three-month follow-up after discharge, the patient remained asymptomatic, with no neurologic deficits on examination and no readmissions or complications.

## Discussion

This case illustrates two important points. First, the patient presented without pain, with transient bilateral lower-limb weakness as the initial symptom. Second, hypotension and bradycardia were noted on arrival. According to Spittell et al., 33 of 235 patients diagnosed with aortic dissection-approximately 15%-presented without pain [1]. Meanwhile, Fatima and Sharma, summarizing IRAD data, reported that in painless dissection, initial non-pain presentations frequently included syncope (33.9%), new-onset neurologic deficits (23.7%), stroke or congestive heart failure (19.7%), and coma or spinal cord ischemia (17.0%) [4]. Cardiac arrhythmias, including bradycardia, have been reported in 11.4% of painless dissections [5]. Although the frequency of hypotension specifically in patients presenting without pain is not well defined, in a study by Tsai et al., in which 1,073 aortic dissection patients were included, hypotension was observed in approximately 29.2% [6].

Regarding the transient lower-limb weakness in our case, the dissection was confined to the aortic arch, and the symptom had completely resolved by the time of examination; therefore, spinal cord hypoperfusion secondary to systemic hypotension is the most plausible mechanism [7]. As this case underscores, aortic dissection may present primarily with neurologic symptoms in the absence of pain [4].

Moreover, aphasia, impaired consciousness, or transient global amnesia (TGA) can prevent patients from reporting chest pain, further complicating diagnosis. When clinicians encounter atypical combinations of neurologic signs, for example, simultaneous involvement of central and peripheral nervous systems or co-occurrence of syncope, seizure, and ischemia of the brain/spinal cord/peripheral nerves, aortic dissection should be considered in the differential. Because neurologic symptoms occur in 17-40% of dissections, a focused neurologic examination should not be neglected even in unstable patients. Importantly, preoperative neurologic deficits alone should not preclude urgent surgery; when aortic dissection is recognized early and treated appropriately, neurologic symptoms do not necessarily translate into increased mortality [8,9].

In addition, although bradycardia with hypotension persisted in our patient, imaging showed no involvement of the coronary ostia or coronary arteries and no pericardial effusion, making bradycardia due to coronary ischemia or cardiac tamponade unlikely. Instead, reflex-mediated bradycardia is suggested via the Bezold-Jarisch reflex, carotid sinus reflex, or aortic baroreceptor reflex [10-12]. The absence of myocardial ischemia or left ventricular abnormality, together with arch and bilateral carotid extension, supports the possibility that baroreceptor stimulation in the aortic arch or carotid sinus triggered vagally mediated bradycardia. Indeed, profound bradycardia and hypotension associated with carotid artery dissection have been reported [13].

From a diagnostic standpoint, TTE for type A dissection shows a sensitivity of 62.4% and specificity of 87.5%, limiting its utility as a stand-alone rule-out test [14]. By contrast, carotid extension occurs in approximately 23% of type A dissections [15], and carotid ultrasonography can detect carotid artery dissection with high sensitivity (~95%) [16]. In our patient, bedside carotid ultrasonography performed during the evaluation of hypotension and bradycardia incidentally visualized an intimal flap, which proved pivotal for rapid diagnosis.

The RUSH (Rapid Ultrasound in Shock) protocol is a point-of-care ultrasound (POCUS) pathway that systematically evaluates the heart, intravascular volume, and major vessels to differentiate cardiogenic, hypovolemic, obstructive, and distributive shock, and it was designed to provide a more detailed assessment than FAST (Focused Assessment with Sonography for Trauma) [17,18]. POCUS refers to a goal-directed, real-time bedside ultrasound examination performed by the treating clinician as an extension of the physical examination to support immediate clinical decision-making in emergency and intensive care settings, including rapid diagnosis, procedural guidance, and monitoring [19-23].

Although we could not find any study that formally combines carotid ultrasonography with FAST, in scenarios where painless aortic dissection is suspected, unexplained hypotension or bradycardia, particularly with transient neurologic deficits, adding carotid scanning as an adjunct may help reduce missed diagnoses. Carotid artery extension of aortic dissection (CAEAD) has long been recognized as a complication. In a Swiss retrospective series, carotid duplex ultrasonography was performed in 39 of 61 patients undergoing surgery for Stanford type A dissection, and carotid artery dissection was identified in 16 (41%) [24]. Nevertheless, because a substantial proportion of dissections do not extend to the carotid arteries, a negative carotid ultrasound does not exclude aortic dissection; definitive imaging (e.g., CECT/CTA) should still be pursued.

## Conclusions

This case demonstrates that painless acute aortic dissection can present solely with transient neurologic symptoms accompanied by bradycardia and hypotension. When unexplained bradycardia or hypotension occurs with neurologic symptoms, clinicians should include painless aortic dissection in the differential and consider adding carotid ultrasonography as an adjunct to a RUSH-based shock assessment to help avoid missed diagnoses.
